# Clinical and epidemiological profile of pediatric patients undergoing extracorporeal membrane oxygenation: experience at a referral center^1^

**DOI:** 10.1590/1518-8345.7999.4780

**Published:** 2026-03-30

**Authors:** Karleandro Pereira do Nascimento, Maria Veraci Oliveira Queiroz, Silvania Braga Ribeiro, Viviane Martins da Silva, Marcos Venícios de Oliveira Lopes, Thais Moreira de Sena

**Affiliations:** 1 Universidade Estadual do Ceará, Centro de Ciências da Saúde, Fortaleza, CE, Brazil.; 2 Scholarship holder at the Fundação Cearense de Apoio ao Desenvolvimento Científico e Tecnológico (FUNCAP), Brazil.; 3 Universidade Federal do Ceará, Faculdade de Farmácia, Odontologia e Enfermagem, Fortaleza, CE, Brazil.

**Keywords:** Nursing, Pediatrics, Medical Records, Advanced Practice Nursing, Critical Care, Extracorporeal Membrane Oxygenation.

## Abstract

**Objective:**

to analyze the clinical-epidemiological profile of pediatric patients receiving extracorporeal membrane oxygenation support at a referral center.

**Method:**

this was a cross-sectional, retrospective study based on the analysis of 108 electronic and physical medical records of pediatric patients from a public hospital. Sociodemographic and clinical data were collected using an instrument developed for this study. For the analysis, absolute and percentage frequencies, measures of central tendency and dispersion, Shapiro-Wilk normality test, Pearson’s chi-square test, logistic regression model with Stepwise input, multiple linear regression analysis, and Durbin-Watson test were used.

**Results:**

of the documents analyzed, 55.5% belonged to female patients. Regarding clinical outcomes, 76.1% were awaiting surgery before cannulation, 69.3% used vasoactive drugs, and 57.4% died. Central venoarterial extracorporeal membrane oxygenation was the most used modality. The main indication for support was low cardiac output syndrome. Patients who developed mechanical complications during extracorporeal membrane oxygenation had a statistically higher risk of death (odds ratio of 2.8).

**Conclusion:**

mechanical complications were associated with an increased risk of mortality. The clinical-epidemiological profile highlights clinical severity, characterized by hemodynamic instability and surgical indication.

## Highlights

**(1)** Central VA ECMO was the most used modality in pediatric patients. **(2)** Low cardiac output syndrome was the main indication for ECMO. **(3)** Mechanical complications increased the risk of death by 2.8 times. **(4)** Predominance of female infants with congenital heart disease. **(5)** Mortality rate of 57.4% among pediatric patients undergoing ECMO.

## Introduction

Extracorporeal membrane oxygenation (ECMO) functions as cardiopulmonary support for cases of heart failure (cardiogenic shock) and/or acute respiratory failure (hypoxemia or hypercapnia) that are refractory to medical treatment but have the potential for recovery. Although referred to as a therapy, ECMO should be understood as temporary support, used as a bridge to recovery, decision-making, or transplant^1^ ^-^ ^2^. Its use in Brazil is relatively recent, being recognized by Opinion 42/2017 of the Federal Council of Medicine as a non-experimental procedure^3^. The country has 21 centers accredited by the Extracorporeal Life Support Organization (ELSO), an international entity that establishes guidelines on this technology^4^.

ECMO represents one of the great advances in modern intensive care medicine for critically ill patients. The individual’s clinical condition and the affected organ determines the type of support offered. The venoarterial (VA) modality offers cardiac support with or without preserved pulmonary function, most frequently indicated for transient post-cardiotomy failure or cardiogenic shock. The venovenous (VV) modality is used for respiratory support in patients with preserved cardiac function, such as in pneumonia^5^ ^-^ ^6^. ECMO also covers other modalities such as E-CPR (extracorporeal cardiopulmonary resuscitation)^7^ and combined configurations such as VVV (veno-veno-venous), VVA (veno-veno-arterial), and VAV (veno-arterial-venous), which address more complex situations^5^.

The first recorded success of the use of an extracorporeal circulation device occurred in heart surgery in 1954. In 1972, the first use of ECMO as therapeutic support in acute respiratory distress syndrome (ARDS) was recorded. The first neonatal case was described in 1975 in a patient with meconium aspiration syndrome^8^. In the pediatric context, the most common indications are preoperative hemodynamic instability, postoperative low cardiac output syndrome, critical congenital heart disease, and inability to wean from cardiopulmonary bypass. Considering the pediatric population with congenital heart disease alone, extracorporeal support is commonly indicated for pre- or post-surgical clinical stabilization and in the E-CPR scenario^9^.

The length of time a patient remains on ECMO varies and can extend for days or weeks. Discontinuation of therapy is related to improvement in the clinical condition that justified the need for support, the absence of prospects for recovery, or the irreversibility of the condition^5^. ECMO in pediatric patients has particularities in management compared to adults, such as the need for specific adjustments in hemodynamic parameters and the higher risk of thrombotic and hemorrhagic complications^2^, aspects that motivated this study.

Analysis of the clinical and epidemiological profile of pediatric patients on ECMO broadens the multidisciplinary team’s understanding of complications during support, prognosis, and outcomes of bridge therapy^2^ ^,^ ^9^. Medical records document the main challenges associated with the use of ECMO, the most prevalent diagnoses that justified its indication, the therapeutic approaches adopted, responses to treatment, and clinical outcomes. These data reflect the continuous assessment of the patient, from cannulation to discharge. Understanding these relationships will contribute to the planning of interventions that minimize failures in care and improve the management of extracorporeal support.

In view of the above, the following guiding question was developed for this study: What is the clinical-epidemiological profile of pediatric patients undergoing ECMO in a referral center? To answer this question, the objective was to analyze the clinical-epidemiological profile of pediatric patients undergoing extracorporeal membrane oxygenation support in a referral center.

## Method

### Type of study

A cross-sectional, retrospective, single-center quantitative study^10^ conducted using electronic and physical medical records of pediatric patients.

### Collection location and period 

Data collection was conducted by two researchers at a high-complexity referral cardiopulmonary hospital located in Fortaleza, CE, Brazil, between May and August 2024. This institution serves patients from pediatric to adult age groups, covering the North and Northeast regions of the country, and is part of one of the largest health complexes fully affiliated with the Unified Health System (SUS). The hospital has eight intensive care units (ICUs), two of which are dedicated to the care of children with congenital heart disease. Among them, one ICU is exclusively for post-operative cardiac surgery and ECMO patients, with eight beds. The study was conducted in this unit.

### Population, selection criteria, and sample

The population consisted of all pediatric patients who underwent ECMO between July 2012, the year and month the program began, and April 2024, totaling 12 years of experience in extracorporeal care at this center. The records of all patients cannulated during this period and admitted to the hospital’s pediatric cardiac surgery postoperative ICU were analyzed. The study used a non-probabilistic convenience sample, resulting in a total of 108 pediatric individuals; there was no neonatal cannulation in the period analyzed. As the sample included all available medical records, no documents were excluded, and therefore there were no exclusion criteria.

### Data collection and study variables

The physical medical records were located in the archives of the Ceará State Health Department and transported to the collection center for data extraction. The electronic medical records were accessed directly on the computers of the originating service, ensuring that data were obtained in a systematic and confidential manner. The physical medical records were included because, at the beginning of ECMO implementation at the study hospital, there were no electronic health records. The medical records were read in a room at the Department of Medical Records and Archives of the study hospital. 

The variables evaluated were: sex, age, weight, primary diagnosis, indication for support, use of vasoactive drugs before ECMO, number of days of invasive mechanical ventilation before support was initiated, occurrence of previous cardiopulmonary arrest (CPA), post-cardiotomy as an indication, physical location of cannulation, ECMO modality, duration of extracorporeal support, use of antibiotics (when applicable), mechanical and physiological complications, and patient outcome.

Mechanical complications are defined as: oxygenator membrane failure; pump failure; circuit rupture; problems with cannulas (misposition or obstruction by clots); and gas embolism. Physiological complications include: hemorrhage; acute kidney injury and/or need for renal replacement therapy; central nervous system injury; electrolyte and metabolic imbalances; cardiac complications, including the use of inotropic/vasopressor agents; and other complications, including infections, pneumothorax, hemolysis, and pressure injury^11^.

### Data processing and analysis

The information collected by the researchers was organized in an Excel spreadsheet stored on a drive. The data were structured based on a collection instrument developed specifically for this research. The medical records were read according to the categories of analysis established in the instrument, respecting the code of ethics of the professions involved and the recording methods adopted by the professionals.

The data were analyzed using absolute and percentage frequencies for qualitative variables, while for quantitative variables, measures of mean and median central tendency and dispersion, standard deviation, and interquartile range were presented. In addition, the Shapiro-Wilk test was used to verify adherence to normal distribution. Pearson’s chi-square test was applied to verify the association between clinical variables and death. 

To identify the characteristics that influenced the type of ECMO, a logistic regression model with Stepwise input was applied. To evaluate the factors that influenced the duration of ECMO, multiple linear regression analysis was used with adjustment verification by the coefficient of determination (R2). The autocorrelation of the residuals was analyzed by the Durbin-Watson test.

### Ethical aspects

All ethical and legal requirements for research involving human subjects were met, with approval from the Research Ethics Committee of the proposing institution, according to opinion number 6,050,790 and CAAE number 69346823.4.0000.5039. Consent was obtained from the hospital where the study was conducted and authorization from the managers of the pediatric cardiac postoperative ICU. The information collected was treated confidentially, ensuring the anonymity of the patients and the confidentiality of the research site, in accordance with regulatory standards^12^.

## Results

Over a period of twelve years (2012-2024), 108 pediatric patients underwent ECMO support. The sample covered all available documents. However, some variables were missing due to a lack of records, especially in physical medical records, which explains the difference in the total values presented in Tables 2 and 3. 

Of the documents analyzed, the majority corresponded to female patients (60; 55.5%). The results show a median age of nine months among the patients, with a median weight of seven kilograms. Of the 108 medical records, 52 were from the physical archive, from the collection and custody of the Ceará State Health Secretariat, while 56 belonged to the electronic patient record (EPR) program, which was started at the hospital in 2020, as shown in Table 1.

**Table 1:** Characterization of the sociodemographic profile of pediatric patients undergoing ECMO* (n = 108). Fortaleza, CE, Brazil, 2025

Variables	n^†^		%^‡^		
Sex					
Female	60		55.56		
Male	48		44.44		
Variables	Mean	Standard deviation	Median	IIQ^§^	p-value^||^
Age (months)	38.0	63.5	9.0	35.0	<0.001
Weight (kg)	15.1	20.1	7.0	9.0	<0.001

*ECMO = Extracorporeal membrane oxygenation; ^†^n = Participants; ^‡^% = Percentage; ^§^IIQ = Interquartile range; ^||^p-value = Null hypothesis

Table 2 shows the clinical outcome of pediatric patients, showing that more than half were awaiting surgery due to the severity of congenital heart disease (64; 76.1%) before cannulation or were using inotropic or vasopressor drugs (52; 69.3%). The most used modality was VA ECMO, predominantly of the central type. Support was mainly installed in the operating room of the hospital of origin, and the main indication for the use of support was low cardiac output syndrome.

**Table 2:** Clinical outcome of pediatric patients, ECMO* modality and type, physical location of installation, and indication for support (n = 108). Fortaleza, CE, Brazil, 2025

Variable			n^†^		%^‡^
Clinic					
Pre-ECMO* surgical correction			64		76.19
Physiological complications			64		73.56
Use of antibiotics on the day of installation			64		73.56
Pre-ECMO* vasoactive drug			52		69.33
Death			54		57.45
Mechanical complications			41		47.13
Pre-ECMO* CPR			28		36.84
ECMO modality*					
Venous-arterial			74		92.50
Venous-venous			6		7.50
Type of ECMO*					
Central			52		65.00
Peripheral			28		35.00
Installation location					
Operating room at the hospital of origin			45		56.25
Intensive care unit at the hospital of origin			33		41.25
Another hospital			2		2.50
ECMO indication*					
Low cardiac output syndrome			22		26.83
Cardiogenic shock			13		15.85
Pulmonary hypertension crisis/Hypoxemia			13		15.85
Acute respiratory distress syndrome/Coronavirus			9		10.98
Other causes			8		9.76
Post-extracorporeal circulation			5		6.10
Right ventricular dysfunction			5		6.10
Pneumonia/Septic shock			4		4.88
Variables	Mean	Standard deviation	Median	IIQ^||^	p-value^¶^
Mechanical ventilation (days)	3.5	4.3	1.0	3.2	<0.001
Extracorporeal membrane oxygenation time	9.5	9.8	6.0	6.0	<0.001

*ECMO = Extracorporeal membrane oxygenation; ^†^n = Participants; ^‡^% = Percentage; ^§^CPR = Cardiopulmonary resuscitation; ^||^IQR = Interquartile range; ^¶^p-value = Null hypothesis

Children who experienced mechanical complications during ECMO had an almost three times higher chance of dying compared to those who did not develop such complications (odds ratio of 2.8). It is important to note that the sample comprised all available documents; however, some variables presented incomplete data, mainly in physical medical records, justifying the total variation in Table 3.

**Table 3:** Mechanical complications and death in pediatric patients on ECMO* (n = 87). Fortaleza, CE, Brazil, 2025

Mechanical complication	Death		Total	χ^2†^	p-value^‡^	Odds Ratio (95%CI^§^)
	Yes	No				
Yes	28	13	41	5.40	0.010	2.80 (1.16 - 6.74)
No	20	26	46			
Total	48	39	87			

*ECMO = Extracorporeal membrane oxygenation; ^†^χ2 = Chi-square; ^‡^p-value = Null hypothesis; ^§^95% CI = Confidence interval

In Table 4, the logistic regression model showed a reduction in the chance of peripheral ECMO use among children who did not present with pre-ECMO CPA and underwent the procedure in the operating room compared to those treated in the ICU. On the other hand, the chance of central ECMO was higher among children in the ICU and with pre-ECMO CPA.

In Table 5, the linear regression model for ECMO duration showed that support time was longer among female children and those who had not undergone surgical correction.

**Table 4:** Multivariate logistic regression model for type of ECMO* (peripheral) (n = 108). Fortaleza, CE, Brazil, 2025

Variables	Coef^†^	SE^‡^	z^§^	Wald^||^	df^¶^	p-value**	Odds Ratio^††^	95%CI^‡‡^
Intercept	1.05	0.73	1.43	2.04	1	0.153		
Age (months)	0.03	0.01	3.10	9.62	1	0.002	1.03	1.01 - 1.04
Pre-ECMO* CPR^§§^ (No)	-1.89	0.78	-2.43	5.92	1	0.015	0.15	0.03 - 0.69
Facility: hospital of origin	-2.76	0.81	-3.40	11.53	1	<0.001	0.06	0.01 - 0.31
Facility: other center	-0.43	1.65	-0.26	0.07	1	0.796	0.65	0.03 - 16.50

*ECMO = Extracorporeal membrane oxygenation; ^†^Coef = Coefficient; ^‡^SE = Standard error; ^§^z = Statistics; ^||^Wald = Wald test; ^¶^df = Degrees of freedom; **p-value = Null hypothesis; ^††^Odds Ratio = Odds Ratio; ^‡‡^95% CI = Confidence Interval; ^§§^CPR = Cardiopulmonary Resuscitation

**Table 5:** Multiple linear regression model for ECMO time*. Fortaleza, CE, Brasil, 2025

Variables				Coef^†^	SE^‡^	t^§^	p-value^||^
Intercept				5.34	1.78	3.00	0.004
Gender (Female)				4.45	2.17	2.05	0.044
Pre-ECMO* surgical correction (No)				6.39	2.44	2.62	0.011
Anova			SQ^¶^	df**	QM^††^	F^‡‡^	p-value^||^
Regression			896.2	2	448.1	5.18	0.008
Residual			6232.4	72	86.6		
Total			7128.7	74			
Adjusted			R²^§§^	R²aj^||||^	Autocorr^¶¶^.	DW***	p-value^||^
			0.126	0.101	-0.041	2.08	0.755

*ECMO = Extracorporeal membrane oxygenation; ^†^Coef = Coefficient; ^‡^SE = Standard Error; ^§^t = T-statistic; ^||^p-value = Null hypothesis; ^¶^SS = Sum of Squares; **df = Degrees of Freedom; ^††^MS = Mean Square; ^‡‡^F = F-statistic; ^§§^R² = Coefficient of Determination; ^||||^R²aj = Adjusted Coefficient of Determination; ^¶¶^Autocorr. = Autocorrelation; ***DW = Durbin-Watson Test.

## Discussion

This study enabled us to analyze the clinical and epidemiological profile of pediatric patients undergoing ECMO at a referral hospital specialized in cardiopulmonary medicine in the state of Ceará. This therapy is considered a bridge when acute pulmonary and/or cardiac failure represents a mortality risk greater than 50% and is strongly indicated when the probability of death reaches about 80%, despite conventional treatment^13^ ^-^ ^14^. In line with what has been described in the literature^15^ ^-^ ^16^, the data from this study point to a predominance in the use of ECMO in female infants, being more frequently indicated for cases of congenital heart disease. The findings are consistent with a previous study that indicates a high prevalence of ECMO in this age group^16^.

The ELSO recommends selection criteria in cases of respiratory failure and heart failure with or without associated pulmonary dysfunction for pediatric patients who will undergo therapy^14^ ^,^ ^17^. Decisions should be made considering the individuality of each patient, based on knowledge of the clinical condition, the experience of the center, the consensus of the multidisciplinary team, and bedside consultation^14^. In this study, the indication was made within the internationally established criteria, with emphasis on the duration of mechanical ventilation, which had an average of 3.5 days until the moment of cannulation. It is observed that survival rates are higher when the period of mechanical ventilation is less than 14 days prior to ECMO^18^ ^-^ ^19^.

Data analysis reveals that patients were already using vasoactive drugs before the start of ECMO, evidencing hemodynamic instability prior to cannulation. In addition, a significant proportion of individuals had CPR before the start of therapy. Although vasoactive drugs can act as hemodynamic rescue for critically ill patients, their use is associated with worse outcomes, such as renal nephrotoxicity and tissue hypoperfusion, longer ICU stays, and increased mortality^20^. During ECMO, the doses of these drugs are usually gradually reduced until they are discontinued, with the need for maintenance or withdrawal assessed on a case-by-case basis^16^.

The use of vasoactive drugs reflects the clinical severity of patients with congenital heart disease awaiting palliative or corrective surgery or hemodynamic procedures. In about one-third of cases, CPR was the main indication for ECMO use. In this situation, VA ECMO may be recommended when the patient does not respond to conventional cardiopulmonary resuscitation (CPR) maneuvers, a procedure known as E-CPR^21^ ^-^ ^22^. Restricted to in-hospital CPR, E-CPR should be instituted during chest compressions or within 20 minutes after the return of spontaneous circulation without continuous compressions. Its use is associated with less favorable neurological outcomes when compared to ECMO used for other indications^22^.

In the present study, patients undergoing cannulation without CPR as a criterion presented physiological complications such as bleeding, pressure injury, infection, and hydroelectrolytic and metabolic imbalance, a trend also reported in a previous study^11^. Mechanical complications such as clot formation in the cannulas, accidental decannulation, and oxygenator failure were also observed in this study, consistent with reports from a previous study^18^. It was found that the underlying disease increased the risk of bleeding and thromboembolism, associated with the use of continuous anticoagulants and the non-biological interface of the circuit, similar to that found in another study^16^. It should be added that centrally predominant VA support, described in most cases, causes impaired cardiac performance, reduced coronary flow, decreased oxygen transport to the brain and vital organs, and increased pulmonary resistance^23^.

VV support is associated with a lower risk of neurological injury and mortality and is recommended as the initial modality for cases of respiratory failure in pediatrics^14^. In this condition, bacterial pneumonia is a frequent cause of ECMO in this age group^24^. 

In this study, the main indications for extracorporeal support were low cardiac output syndrome, cardiogenic shock, and pulmonary hypertension/hypoxemia crisis, with a mortality rate exceeding half of the analyzed population. Given the predominance of cardiac causes, it is speculated that factors such as the use of VA ECMO, prior use of inotropes and vasopressors, timely indication of support, underlying disease, and even demographic aspects such as age group may have influenced the observed mortality.

Studies indicate that indication based on well-defined criteria contributes to the success of therapy^13^ ^,^ ^15^ ^,^ ^25^. The goal of ECMO is not curative, but to provide support to critically ill patients when conventional options prove ineffective, increasing their chances of survival^17^. Unlike other methods, such as ventricular assist devices (VADs), which require installation in a sterile environment, ECMO can be implemented at the bedside^26^, as was the case in this study. 

Although extracorporeal support is a vital strategy for critically ill patients, the presence of multiple connectors and access points increases the risk of complications and operational challenges, such as bleeding and thrombus formation in the circuit, both of which are associated with a high mortality rate^23^ ^,^ ^25^. In this study, the main causes of death were multiple organ failure, CPA, and septic shock.

Prolonged connection of the body to the polyvinyl surface of the cannulas, the use of multiple medical devices, and the individual’s clinical condition represent risk factors for infections. The handling of this patient by the multidisciplinary team, using aseptic techniques, is essential to minimize this risk^26^ ^-^ ^27^. Although the literature does not present conclusive data associating the site of ECMO installation with an increased risk of infection, the adoption of asepsis is mandatory, especially when cannulation occurs in the ICU^28^. The data show that patients were already using antibiotics before cannulation, which reflects the complexity of the clinical-infectious picture. The average duration of ECMO use was 9.5 days, similar to results reported in the literature^15^ ^-^ ^16^.

Despite advances in device management, the risks associated with prolonged use of support remain inevitable^29^. It was observed that children who developed mechanical complications had a significantly higher risk of death, almost three times higher than those who did not have these complications. The most common complications include poor cannula positioning, accidental decannulation, clot formation in the oxygenator, and inadvertent removal of cannulas by awake patients^11^ ^,^ ^29^ ^-^ ^30^. Continuous monitoring and rapid response to adverse events during ECMO are essential. Although rare, these complications can cause irreversible damage to the patient or even death^30^.

In this study, accidental decannulation occurred in one patient during a change in position, performed by two professionals to prevent pressure injury. However, the radiological image already indicated a high risk for this adverse event, which was not previously identified by the team. Considered catastrophic, accidental decannulation can cause massive bleeding and air entry into the circuit^15^ ^,^ ^30^. This was the most serious adverse event in the sample, which led to the implementation of training and preventive strategies. The patient died due to the underlying disease, not decannulation, in another hospital unit.

This study reveals a lower probability of peripheral ECMO use in patients without CPA before cannulation and who had undergone cardiac surgery. Support was more often needed in patients admitted to the ICU with previous CPA who had not undergone surgery. These data highlight the importance of timely interventions, since children with congenital heart disease, who comprised almost the entire sample, require surgical procedures at specific ages, according to the physiology of the underlying disease^15^ ^,^ ^20^. Due to the severity of these conditions, the use of ECMO is common in critically ill congenital heart disease patients^16^.

Although the management of extracorporeal support involves a multidisciplinary team, ELSO highlights the role of nursing in this process. As it is a highly complex and costly therapy, the responsibility for direct care of patients on ECMO, within the nursing team, is exclusive to nurses^31^. With the implementation of the Nursing Process (NP) and institutional protocols, it is possible to ensure systematic and safe care^32^, in line with ELSO’s recommendation to maintain a 1:1 ratio between nurses and patients on ECMO^33^. It is imperative that professionals have the fundamental knowledge, skills, and attitudes to manage these patients^27^ ^,^ ^34^.

Finally, it is important to note that the hospital in the study underwent a significant transition in its records. Until September 2020, notes were made in physical medical records. After this period, the EPR was implemented, and in 2023, the NP and the Systematization of Nursing Care (SAE), previously based on physical checklists, became fully digital. In addition, the other members of the multidisciplinary health team also adopted the same recording platform, reducing the time spent on notes and allowing more time for patient care.

The limitation of this study is that it is based on records that do not include long-term outcomes. However, during the collection of variables, the researchers noted the telephone numbers of those responsible for the patients, with the aim of assessing the disabilities and quality of life of survivors in the future through telemedicine. Another limitation was the lack of complete information in some medical records, especially in physical records, such as the modality and type of ECMO used. The fact that the research was conducted at only one center limits generalizations to other contexts. 

In addition to the research, the data will be shared with the hospital where the study was conducted, which may assist the multidisciplinary team in the timely selection of candidates for extracorporeal support, increasing the chances of success of bridge therapy and optimizing the center’s resources. The study shows that the use of ECMO and the clinical conditions of patients are associated with the occurrence of adverse events, such as mechanical and physiological complications. Given the scarcity of studies in the pediatric age group undergoing ECMO, this research makes significant contributions to the topic, representing an advance in scientific knowledge in the health field, in the analysis and dissemination of little-explored subjects. 

For nursing, knowledge of the clinical-epidemiological profile allows nurses to understand the particularities of this population, adapt care based on the patient’s clinical condition and the type of ECMO offered, strengthening evidence-based practice and safe and effective care. 

## Conclusion

Mechanical complications in ECMO were significantly associated with an increased risk of mortality among the patients analyzed. The clinical-epidemiological profile highlights the severity of the clinical condition, marked especially by the presence of hemodynamic instability and the need for surgical intervention. In addition, the prior use of vasoactive drugs and antibiotics prior to cannulation reflects cardiac and infectious deterioration, thereby contributing to the high mortality observed in this population.

This study contributes to filling gaps in clinical practice in pediatric patients undergoing ECMO, serving as a basis for the implementation of continuing education programs and the development of specific lines of care for this population. The importance of new clinical and epidemiological research for a more comprehensive characterization of pediatric patients who are candidates for ECMO, in addition to long-term monitoring of survivors, should be emphasized.

## Data Availability

All data generated or analysed during this study are included in this published article.
