# Supplementary material for: Clinical and epidemiological profile of pediatric patients undergoing extracorporeal membrane oxygenation: experience at a referral center
Source: Rev Lat Am Enfermagem. 2026 Mar 30;34:e4780. doi: 10.1590/1518-8345.7999.4780 (PMC13035204; doi:10.1590/1518-8345.7999.4780)
Supplement: Translation

# Translation: Perfil clínico-epidemiológico de pacientes pediátricos em oxigenação por membrana extracorpórea: experiência de um centro^2^

**DOI:** 10.1590/1518-8345.7999.4781

**Authors:** Karleandro Pereira do Nascimento, Maria Veraci Oliveira Queiroz, Silvania Braga Ribeiro, Viviane Martins da Silva, Marcos Venícios de Oliveira Lopes, Thais Moreira de Sena

**Affiliations:** 1 Universidade Estadual do Ceará, Centro de Ciências da Saúde, Fortaleza, CE, Brazil.; 2 Scholarship holder at the Fundação Cearense de Apoio ao Desenvolvimento Científico e Tecnológico (FUNCAP), Brazil.; 3 Universidade Federal do Ceará, Faculdade de Farmácia, Odontologia e Enfermagem, Fortaleza, CE, Brazil.

**Keywords:** Enfermagem, Pediatria, Prontuários Médicos, Prática Avançada de Enfermagem, Cuidados Críticos, Oxigenação por Membrana Extracorpórea.

## Abstract

**Objetivo:**

analisar o perfil clínico-epidemiológico de pacientes pediátricos em suporte por oxigenação por membrana extracorpórea em um centro de referência.

**Método:**

estudo transversal e retrospectivo, baseado na análise de 108 prontuários de pacientes pediátricos de um hospital público. Foram coletados dados sociodemográficos e clínicos por meio de um instrumento desenvolvido para esta pesquisa. Para a análise, foram utilizadas frequências absolutas e percentuais, medidas de tendência central e dispersão, teste de normalidade de Shapiro-Wilk, teste qui-quadrado de Pearson, modelo de regressão logística com entrada *Stepwise*, análise de regressão linear múltipla e teste de Durbin-Watson.

**Resultados:**

segundo a análise dos documentos, 55,5% pertenciam a pacientes do sexo feminino. Quanto aos desfechos clínicos, 76,1% aguardavam cirurgia antes da canulação, 69,3% utilizaram drogas vasoativas e 57,4% evoluíram para óbito. A oxigenação por membrana extracorpórea venoarterial do tipo central foi a modalidade mais utilizada. A principal indicação para o suporte foi a síndrome do baixo débito cardíaco. Pacientes que desenvolveram complicações mecânicas na oxigenação por membrana extracorpórea apresentaram risco estatisticamente maior para o óbito (*odds ratio* de 2,8).

**Conclusão:**

complicações mecânicas se associaram ao risco aumentado de mortalidade. O perfil clínico-epidemiológico evidencia a gravidade clínica, caracterizada por instabilidade hemodinâmica e indicação cirúrgica.

## Destaques

**(1)** ECMO VA central foi a modalidade mais utilizada nos pacientes pediátricos. **(2)** A síndrome do baixo débito cardíaco foi a principal indicação para ECMO. **(3)** As complicações mecânicas aumentaram em 2,8 vezes o risco de óbito. **(4)** Predominância de lactentes do sexo feminino com cardiopatias congênitas. **(5)** Mortalidade de 57,4% entre pacientes pediátricos submetidos à ECMO.

## Introdução

A oxigenação por membrana extracorpórea (ECMO) funciona como um suporte cardiopulmonar para quadros de insuficiência cardíaca (choque cardiogênico) e/ou respiratória (hipoxemia ou hipercapnia) aguda e refratária aos tratamentos médicos, mas com potencial de recuperação. Embora referida como uma terapia, a ECMO deve ser entendida como um suporte temporário, utilizado como ponte para recuperação, tomada de decisão ou transplante^1^ ^-^ ^2^. Sua utilização no Brasil é relativamente recente, sendo reconhecida por meio do parecer 42/2017 do Conselho Federal de Medicina como um procedimento não experimental^3^. O país conta com 21 centros credenciados na *Extracorporeal Life Support Organization* (ELSO), uma entidade internacional que estabelece diretrizes e *guidelines* sobre essa tecnologia^4^.

A ECMO representa um dos grandes progressos da medicina intensiva moderna para pacientes em estado crítico. A condição clínica do indivíduo e o órgão acometido determinam o tipo de suporte ofertado. A modalidade venoarterial (VA) oferece suporte cardíaco com função pulmonar preservada ou não, tendo como indicação mais frequente a falência transitória pós-cardiotomia ou choque cardiogênico. A modalidade venovenosa (VV) é utilizada para suporte respiratório em pacientes com função cardíaca preservada, como nas pneumonias^5^ ^-^ ^6^. A ECMO também abrange outras modalidades como a E-CPR (ressuscitação cardiopulmonar extracorpórea)^7^ ^)^ e as configurações combinadas como a VVV (veno-veno-venosa), VVA (veno-veno-arterial) e VAV (veno-arterial-venosa) que atendem a situações mais complexas^5^.

O primeiro sucesso registrado do uso de um dispositivo de circulação extracorpórea ocorreu em uma cirurgia cardíaca em 1954. Em 1972, foi registrado o primeiro uso da ECMO como suporte terapêutico na síndrome do desconforto respiratório agudo (SDRA). Já o primeiro caso neonatal foi descrito em 1975 em uma paciente com síndrome de aspiração meconial^8^.

No contexto pediátrico, as indicações mais comuns consistem na instabilidade hemodinâmica pré-operatória, na síndrome do baixo débito cardíaco pós-operatório, na doença cardíaca congênita crítica e na incapacidade de se desvencilhar do *bypass* cardiopulmonar. Considerando isoladamente a população pediátrica com doença cardíaca congênita, o suporte extracorpóreo é comumente indicado para a estabilização clínica pré ou pós-cirurgia e no cenário de E-CPR^9^.

O tempo em que o paciente permanece em ECMO é variável, podendo estender-se por dias ou semanas. A interrupção da terapia está relacionada à melhora da condição clínica que justificou a necessidade do suporte, à ausência de perspectiva de recuperação ou à irreversibilidade do quadro^5^. A ECMO em pacientes pediátricos apresenta particularidades no manejo em relação aos adultos, como a necessidade de ajustes específicos nos parâmetros hemodinâmicos e o maior risco de complicações trombóticas e hemorrágicas^2^, aspectos que motivaram a realização deste estudo.

A análise do perfil clínico-epidemiológico de pacientes pediátricos em ECMO amplia a compreensão da equipe multiprofissional sobre complicações durante o suporte, o prognóstico e os desfechos da terapia-ponte^2^ ^,^ ^9^. Os registros nos prontuários documentam os principais desafios associados ao uso da ECMO, os diagnósticos mais prevalentes que justificaram sua indicação, as condutas terapêuticas adotadas, as respostas ao tratamento e os desfechos clínicos. Esses dados refletem a avaliação contínua do paciente, desde a canulação até a alta médica. Entender essas relações contribuirá para o planejamento de intervenções que minimizem falhas na assistência e aprimorem o manejo do suporte extracorpóreo.

Diante do exposto, elaborou-se a seguinte pergunta norteadora para este estudo: Qual é o perfil clínico-epidemiológico de pacientes pediátricos submetidos à ECMO em um centro de referência? Para respondê-la, estabeleceu-se como objetivo analisar o perfil clínico-epidemiológico de pacientes pediátricos em suporte da oxigenação por membrana extracorpórea em um centro de referência.

## Método

### Tipo do estudo

Estudo transversal, retrospectivo, unicêntrico de base quantitativa^10^, realizado com prontuários eletrônicos e físicos de pacientes pediátricos.

### Local e período da coleta 

A coleta de dados foi realizada por dois pesquisadores em um hospital referência em cardiopneumologia de alta complexidade localizado em Fortaleza, CE, Brasil, entre maio e agosto de 2024. Essa instituição atende pacientes desde a faixa etária pediátrica à idade adulta, abrangendo as regiões Norte e Nordeste do país e integrando um dos maiores complexos de saúde totalmente conveniados pelo Sistema Único de Saúde (SUS). O hospital conta com oito unidades de terapia intensiva (UTI), sendo duas destinadas ao atendimento de crianças com cardiopatias congênitas. Dentre elas, uma UTI é exclusiva para o pós-operatório de cirurgia cardíaca e para pacientes em ECMO, dispondo de oito leitos. O estudo foi conduzido nessa unidade.

### População, critérios de seleção e amostra

A população foi composta por todos os pacientes pediátricos submetidos à ECMO entre julho de 2012, ano e mês de início do programa, e abril de 2024, totalizando 12 anos de experiência em assistência extracorpórea nesse centro. Foram analisados os registros de todos os pacientes canulados nesse período, admitidos na UTI pós-operatória de cirurgia cardíaca pediátrica do hospital. A pesquisa utilizou uma amostra não probabilística por conveniência, resultando em um total de 108 indivíduos pediátricos. Não houve canulação neonatal no período analisado. Como a amostra incluiu todos os prontuários disponíveis, nenhum documento foi excluído, não havendo, portanto, critério de exclusão.

### Coleta de dados e variáveis do estudo

Os prontuários físicos foram localizados no arquivo da Secretaria de Saúde do Estado do Ceará e transportados ao centro de coleta para a extração das informações. Já os prontuários eletrônicos foram acessados diretamente nos computadores do serviço de origem, garantindo a obtenção dos dados de maneira sistemática e sigilosa. Os prontuários físicos foram incluídos, visto que no início da implementação da ECMO no hospital do estudo não havia registros eletrônicos em saúde. A leitura dos prontuários foi feita em uma sala do Departamento de Registro e Arquivos Médicos do hospital do estudo. 

As variáveis avaliadas foram: sexo, idade, peso, diagnóstico primário, indicação para o suporte, uso de droga vasoativa antes da ECMO, número de dias de ventilação mecânica invasiva antes da instalação do suporte, ocorrência de parada cardiorrespiratória (PCR) prévia, pós-cardiotomia como indicação, local físico da canulação, modalidade da ECMO, duração da assistência extracorpórea, uso de antibióticos (quando aplicável), complicações mecânicas e fisiológicas e desfecho do paciente.

Entende-se por complicações mecânicas: falência da membrana oxigenadora; falência da bomba; rotura dos circuitos; problemas com as cânulas (mau posicionamento ou obstrução por coágulos); e embolia gasosa. As complicações fisiológicas incluem: hemorragia; lesão renal aguda e/ou necessidade de terapêutica de substituição renal; lesão do sistema nervoso central; desequilíbrios hidroeletrolítico e metabólico; complicações cardíacas, incluindo o uso de agentes inotrópicos/vasopressores; e outras complicações, incluindo infeções, pneumotórax, hemólise e lesão por pressão^11^.

### Tratamento e análise dos dados

As informações coletadas pelos pesquisadores foram organizadas em uma planilha do *Excel* armazenada em um *drive*. Os dados foram estruturados com base em um instrumento de coleta desenvolvido especificamente para esta pesquisa. A leitura dos prontuários seguiu as categorias de análise estabelecidas no instrumento, respeitando o código de ética das profissões envolvidas e os modos de registro adotados pelos profissionais.

Os dados foram analisados por meio das frequências absolutas e percentuais para variáveis qualitativas, enquanto para variáveis quantitativas foram apresentadas as medidas de tendência central média e mediana, e de dispersão, desvio-padrão e intervalo interquartílico. Além do teste de Shapiro-Wilk para verificação de aderência à distribuição normal. O teste de qui-quadrado de Pearson foi aplicado para verificar a associação entre as variáveis clínicas e o óbito. 

Para identificar as características que influenciavam o tipo de ECMO, foi aplicado um modelo de regressão logística com entrada *Stepwise*. Para avaliação dos fatores que influenciavam no tempo da ECMO, utilizou-se a análise de regressão linear múltipla com verificação de ajuste pelo coeficiente de determinação (R^2^). A autocorrelação dos resíduos foi analisada pelo teste de Durbin-Watson.

### Aspectos éticos

Todos os requisitos éticos e legais para pesquisa com seres humanos foram atendidos, com aprovação do Comitê de Ética em Pesquisa da instituição proponente, conforme parecer de número 6.050.790 e CAAE de número 69346823.4.0000.5039. Foi obtido o termo de anuência do hospital onde o estudo foi conduzido e a autorização dos gestores da UTI pós-operatória cardíaca pediátrica. As informações coletadas foram tratadas com sigilo, assegurando o anonimato dos pacientes e a confidencialidade do local da pesquisa, em conformidade com as normas reguladoras^12^.

## Resultados

Ao longo de um período de doze anos (2012-2024), 108 pacientes pediátricos foram submetidos ao suporte da ECMO. A amostra abrangeu a totalidade dos documentos disponíveis. No entanto, identificou-se ausência de dados em algumas variáveis por falta de registros, sobretudo nos prontuários físicos, o que explica a diferença nos valores totais apresentados nas Tabelas 2 e 3. 

Dos documentos analisados, a maioria correspondia a pacientes do sexo feminino (60; 55,5%). Os resultados demonstram uma idade mediana de nove meses dos indivíduos, com uma mediana de peso de sete quilos. Dos 108 prontuários, 52 eram do arquivo físico, provenientes do acervo e guarda da Secretaria de Saúde do Estado do Ceará, enquanto 56 pertenciam ao prontuário eletrônico do paciente (PEP), programa iniciado no hospital no ano de 2020, conforme Tabela 1. 

A Tabela 2 apresenta o desfecho clínico dos pacientes pediátricos, evidenciando que mais da metade aguardavam cirurgia devido à gravidade da doença cardíaca congênita (64; 76,1%) antes da canulação ou faziam uso de drogas inotrópicas ou vasopressoras (52; 69,3%). A modalidade mais utilizada foi a ECMO VA com predominância do tipo central. O suporte foi majoritariamente instalado no centro cirúrgico do hospital de origem e a principal indicação para o uso do suporte foi a síndrome do baixo débito cardíaco.

**Tabela 1:** Caracterização do perfil sociodemográfico dos pacientes pediátricos submetidos à ECMO* (n = 108). Fortaleza, CE, Brasil, 2025

Variáveis	n^†^		%^‡^		
Sexo					
Feminino	60		55,56		
Masculino	48		44,44		
Variáveis	Média	Desvio-padrão	Mediana	IIQ^§^	Valor p^||^
Idade (meses)	38,0	63,5	9,0	35,0	<0,001
Peso (quilo)	15,1	20,1	7,0	9,0	<0,001

*ECMO = Oxigenação por membrana extracorpórea; ^†^n = Participantes; ^‡^% = Porcentagem; ^§^IIQ = Intervalo Interquartílico; ^||^Valor p = Hipótese Nula

**Tabela 2:** Desfecho clínico dos pacientes pediátricos, modalidade e tipo de ECMO*, local físico da instalação e indicação do suporte (n = 108). Fortaleza, CE, Brasil, 2025

Variáveis			n^†^		%^‡^
Clínica					
Correção cirúrgica pré-ECMO*			64		76,19
Complicações fisiológicas			64		73,56
Uso de antibióticos no dia da instalação			64		73,56
Droga vasoativa pré-ECMO*			52		69,33
Óbito			54		57,45
Complicações mecânicas			41		47,13
PCR^§^ pré-ECMO*			28		36,84
Modalidade da ECMO*					
Venoarterial			74		92,50
Venovenosa			6		7,50
Tipo de ECMO*					
Central			52		65,00
Periférica			28		35,00
Local de instalação					
Centro cirúrgico do hospital de origem			45		56,25
Unidade de terapia intensiva do hospital de origem			33		41,25
Outro hospital			2		2,50
Indicação da ECMO*					
Síndrome do baixo débito cardíaco			22		26,83
Choque cardiogênico			13		15,85
Crise de hipertensão pulmonar/Hipoxemia			13		15,85
Síndrome do desconforto respiratório agudo/Coronavírus			9		10,98
Outras causas			8		9,76
Pós-circulação extracorpórea			5		6,10
Disfunção de ventrículo direito			5		6,10
Pneumonia/Choque séptico			4		4,88
Variáveis	Média	Desvio-padrão	Mediana	IIQ^||^	Valor p^¶^
Ventilação Mecânica (dias)	3,5	4,3	1,0	3,2	<0,001
Tempo de oxigenação por membrana extracorpórea	9,5	9,8	6,0	6,0	<0,001

*ECMO = Oxigenação por membrana extracorpórea; ^†^n = Participantes; ^‡^% = Porcentagem; ^§^PCR = Parada cardiorrespiratória; ^||^IIQ = Intervalo Interquartílico; ^¶^Valor p = Hipótese Nula

Crianças que apresentaram complicações mecânicas durante a ECMO tiveram uma chance quase três vezes maior de evoluírem para o óbito, em comparação àquelas que não desenvolveram tais complicações (*odds ratio de* 2,8). É importante ressaltar que a amostra compreendeu todos os documentos disponíveis, contudo, algumas variáveis apresentaram dados incompletos principalmente nos prontuários físicos, justificando a variação total na Tabela 3. 

**Tabela 3:** Complicação mecânica e óbito dos pacientes pediátricos em ECMO* (n = 87). Fortaleza, CE, Brasil, 2025

Complicação mecânica	Óbito		Total	χ^2†^	Valor p^‡^	***Odds Ratio* (IC - 95%** ^§^)
	Sim	Não				
Sim	28	13	41	5,40	0,010	2,80 (1,16 - 6,74)
Não	20	26	46			
Total	48	39	87			

*ECMO = Oxigenação por membrana extracorpórea; ^†^χ^2^ = Qui-quadrado; ^‡^Valor p = Hipótese Nula; ^§^IC-95% = Intervalo de Confiança

Na Tabela 4, o modelo de regressão logística mostrou uma redução na chance de uso de ECMO periférica entre crianças que não apresentaram PCR pré-ECMO e foram submetidas ao procedimento no centro cirúrgico em comparação com aquelas atendidas na UTI. Por outro lado, a chance de ECMO central era maior entre crianças da UTI e com PCR pré-ECMO.

Na Tabela 5 o modelo de regressão linear para a duração da ECMO demonstrou que o tempo de suporte era maior entre crianças do sexo feminino e que não haviam sido submetidas à correção cirúrgica.

**Tabela 4:** Modelo de regressão logística multivariada para tipo de ECMO* (periférica) (n = 108). Fortaleza, CE, Brasil, 2025

Variáveis	Coef^†^	EP^‡^	z^§^	Wald^||^	gl^¶^	Valor p**	*Odds Ratio* ^*††*^	IC-95%^‡‡^
Intercepto	1,05	0,73	1,43	2,04	1	0,153		
Idade (meses)	0,03	0,01	3,10	9,62	1	0,002	1,03	1,01 - 1,04
PCR^§§^ pré ECMO* (Não)	-1,89	0,78	-2,43	5,92	1	0,015	0,15	0,03 - 0,69
Instalação: hospital de origem	-2,76	0,81	-3,40	11,53	1	<0,001	0,06	0,01 - 0,31
Instalação: outro centro	-0,43	1,65	-0,26	0,07	1	0,796	0,65	0,03 - 16,50

*ECMO = Oxigenação por membrana extracorpórea; ^†^Coef = Coeficiente; ^‡^EP = Erro-Padrão; ^§^z = Estatística; ^||^Wald = Teste Wald; ^¶^gl = Graus de Liberdade; **Valor p = Hipótese Nula; ^††^ *Odds Ratio* = Razão de Chance; ^‡‡^IC-95% = Intervalo de Confiança; ^§§^PCR = Parada Cardiorrespiratória

**Tabela 5:** Modelo de regressão linear múltipla para tempo de ECMO*. Fortaleza, CE, Brasil, 2025

Variáveis		Coef^†^	EP^‡^	t^§^	Valor p^||^
Intercepto		5,34	1,78	3,00	0,004
Sexo (Feminino)		4,45	2,17	2,05	0,044
Correção cirúrgica pré ECMO* (Não)		6,39	2,44	2,62	0,011
Anova	SQ^¶^	gl**	QM^††^	F^‡‡^	Valor p^||^
Regressão	896,2	2	448,1	5,18	0,008
Resíduo	6232,4	72	86,6		
Total	7128,7	74			
Ajuste	R²^§§^	R²aj^||||^	Autocorr^¶¶^.	DW***	Valor p^||^
	0,126	0,101	-0,041	2,08	0,755

*ECMO = Oxigenação por membrana extracorpórea; ^†^Coef = Coeficiente; ^‡^EP = Erro-Padrão; ^§^t = Estatística T; ^||^Valor p = Hipótese Nula; ^¶^SQ = Soma de Quadrados; **gl = Graus de Liberdade; ^††^QM = Quadrado Médio; ^‡‡^F = Estatística F; ^§§^R² = Coeficiente de Determinação; ^||||^R²aj = Coeficiente de Determinação Ajustado; ^¶¶^Autocorr. = Autocorrelação; ***DW = Teste de Durbin-Watson

## Discussão

Por meio deste estudo foi possível analisar o perfil clínico-epidemiológico de pacientes pediátricos com ECMO em um hospital de referência em cardiopneumologia no Estado do Ceará. Considera-se essa terapia-ponte quando a insuficiência da função pulmonar e/ou cardíaca aguda representam risco de mortalidade superior a 50%, sendo fortemente indicada quando a probabilidade de morte atinge cerca de 80%, apesar do tratamento convencional^13^ ^-^ ^14^. Em consonância com o que tem sido descrito na literatura^15^ ^-^ ^16^, os dados desta pesquisa apontam predominância no uso da ECMO em pacientes lactentes do sexo feminino, sendo mais frequentemente indicada para os casos de cardiopatias congênitas. Os achados estão em consonância com estudo prévio que indica uma alta prevalência da ECMO nesse grupo etário^16^.

A ELSO recomenda os critérios de seleção em casos de falência respiratória e de falência cardíaca com ou sem disfunção pulmonar associada, para os pacientes pediátricos que serão submetidos à terapia^14^ ^,^ ^17^. As decisões devem ser tomadas considerando a individualidade de cada paciente, com base no conhecimento da condição clínica, na experiência do centro, no consenso da equipe multiprofissional e em consulta à beira do leito^14^. Neste estudo, a indicação aconteceu dentro dos critérios estabelecidos internacionalmente com destaque para a duração da ventilação mecânica, que apresentou uma média de 3,5 dias até o momento da canulação. Observa-se que as taxas de sobrevivência são maiores quando o período de ventilação mecânica é inferior a 14 dias antes da ECMO^18^ ^-^ ^19^.

A análise dos dados revela que os pacientes já faziam uso de drogas vasoativas antes do início da ECMO, evidenciando um quadro de instabilidade hemodinâmica prévio à canulação. Além disso, uma parcela significativa dos indivíduos apresentou PCR antes do início da terapia. Embora as drogas vasoativas possam atuar como resgate hemodinâmico do paciente crítico, seu uso está associado a piores desfechos, como nefrotoxicidade renal e hipoperfusão tecidual, maior tempo de permanência na UTI e aumento da mortalidade^20^. Durante a ECMO, esses medicamentos geralmente têm suas doses reduzidas de forma gradativa até a suspensão, com a necessidade de manutenção ou retirada avaliada caso a caso^16^.

O uso de drogas vasoativas reflete o agravo clínico dos pacientes com cardiopatias congênitas que aguardavam cirurgias paliativas, corretivas ou procedimentos hemodinâmicos. Em cerca de um terço dos casos, a PCR foi a principal indicação para o uso da ECMO. Nessa situação, a instalação da ECMO VA pode ser recomendada quando o paciente não responde às manobras convencionais de ressuscitação cardiopulmonar (RCP), procedimento conhecido como E-CPR^21^ ^-^ ^22^. Restrita a PCR intra-hospitalar, a E-CPR deve ser instituída durante as compressões cardíacas ou dentro de 20 minutos após o retorno da circulação espontânea sem compressões contínuas. Sua utilização está associada a desfechos neurológicos menos favoráveis quando comparada à ECMO empregada para outras indicações^22^.

No presente estudo, os pacientes submetidos à canulação, sem PCR como critério, apresentaram complicações fisiológicas, como sangramento, lesão por pressão, infecção e desequilíbrio hidroeletrolítico e metabólico, tendência igualmente relatada em estudo prévio^11^. Também foram observadas nesta pesquisa complicações mecânicas como formação de coágulos nas cânulas, decanulação acidental e falha do oxigenador, em consonância com relatos de estudo anterior^18^. Viu-se que a doença de base aumentou o risco de sangramento e de tromboembolismo, associado ao uso do anticoagulante contínuo e a interface não biológica do circuito dado semelhante ao encontrado em outra pesquisa^16^. Cumpre acrescentar que o suporte VA de predominância central, descrito na maioria dos casos, acarreta prejuízos no desempenho cardíaco, redução do fluxo coronariano, menor transporte de oxigênio ao cérebro e aos órgãos vitais e aumento da resistência pulmonar^23^.

O suporte VV está associado a menor risco de lesão neurológica e mortalidade, sendo recomendado como modalidade inicial para os casos de insuficiência respiratória em pediatria^14^. Nessa condição, a pneumonia bacteriana figura como uma causa frequente de ECMO nesse grupo etário^24^. 

Neste estudo, as principais indicações para o suporte extracorpóreo foram a síndrome do baixo débito cardíaco, o choque cardiogênico e a crise de hipertensão pulmonar/hipoxemia, com uma taxa de mortalidade superior à metade da população analisada. Dado o predomínio de causas cardíacas, especula-se que os fatores como a utilização da ECMO na modalidade VA, o uso prévio de inotrópicos e vasopressores, a indicação do suporte em tempo oportuno, a doença subjacente e até mesmo os aspectos demográficos, como a faixa etária, possam ter influenciado a mortalidade observada.

Estudos apontam que a indicação baseada em critérios bem definidos contribui para o sucesso da terapia^13^ ^,^ ^15^ ^,^ ^25^. O objetivo da ECMO não é curativo, mas de oferecer suporte ao paciente crítico quando as opções convencionais se mostram ineficazes, aumentando suas chances de sobrevivência^17^. Diferente de outros métodos, como o dispositivo de assistência ventricular (VAD), que requer instalação em ambiente estéril, a ECMO pode ser implantada à beira do leito^26^, como ocorreu neste estudo. 

Embora o suporte extracorpóreo seja uma estratégia vital para os pacientes em estado crítico, a presença de múltiplos conectores e pontos de acesso aumenta o risco de complicações e desafios operacionais, como sangramento e formação de trombos no circuito, ambos associados a uma alta taxa de mortalidade^23^ ^,^ ^25^. Neste estudo, as principais causas de óbito foram falência de múltiplos órgãos, PCR e choque séptico.

A conexão prolongada do organismo à superfície de polivinila das cânulas, o uso de múltiplos dispositivos médicos e a condição clínica do indivíduo representam fatores de risco para infecções. O manuseio do paciente pela equipe multiprofissional, utilizando técnicas assépticas, é essencial para minimizar este risco^26^ ^-^ ^27^. Embora a literatura não apresente dados conclusivos que associem o local de instalação da ECMO ao aumento do risco de infecção, é mandatória a adoção da assepsia, sobretudo quando a canulação ocorrer na UTI^28^. Os dados mostram que os pacientes já estavam em uso de antibióticos antes da canulação, o que reflete a complexidade do quadro clínico-infeccioso. O tempo médio de utilização da ECMO foi de 9,5 dias, resultados semelhantes aos da literatura^15^ ^-^ ^16^.

Apesar dos avanços no gerenciamento do dispositivo, os riscos associados ao uso prolongado do suporte permanecem inevitáveis^29^. Observou-se que crianças que desenvolveram complicações mecânicas apresentaram um risco de óbito significativamente maior, sendo quase três vezes superior ao das que não tiveram essas complicações. As mais comuns incluem má posição das cânulas, decanulação acidental, formação de coágulos no oxigenador e a remoção inadvertida das cânulas por pacientes despertos^11^ ^,^ ^29^ ^-^ ^30^. A vigilância contínua e a resposta rápida a eventos adversos com ECMO é indispensável. Embora raras, essas complicações podem causar danos irreversíveis ao paciente ou até mesmo o óbito^30^.

Neste estudo, a decanulação acidental ocorreu em um paciente durante a mudança de decúbito, realizada por dois profissionais para prevenir lesão por pressão. No entanto, a imagem radiológica já indicava um alto risco para esse evento adverso, que não foi identificado previamente pela equipe. Considerada catastrófica, a decanulação acidental pode causar sangramento maciço e entrada de ar no circuito^15^ ^,^ ^30^. Esse foi o evento adverso mais grave da amostra, o que motivou a implementação de capacitações e estratégias preventivas. O paciente evoluiu para óbito devido à doença subjacente, e não pela decanulação, em outra unidade hospitalar.

Este estudo revela uma menor probabilidade de utilização da ECMO periférica em pacientes sem PCR antes da canulação e que haviam passado por cirurgia cardíaca. O suporte foi mais frequentemente necessário em pacientes internados na UTI com PCR prévia e que não haviam realizado cirurgia. Esses dados destacam a importância de intervenções oportunas, já que as crianças com cardiopatias congênitas, que compuseram quase toda a amostra, necessitam de procedimentos cirúrgicos em idades específicas, conforme a fisiologia da doença-base^15^ ^,^ ^20^. Devido à gravidade dessas condições, é comum o uso da ECMO nos cardiopatas congênitos críticos^16^.

Embora o manejo do suporte extracorpóreo envolva uma equipe multiprofissional, a ELSO destaca o papel da enfermagem nesse processo. Sendo uma terapia de alta complexidade e elevado custo, a responsabilidade pela assistência direta aos pacientes em ECMO, dentro da equipe de enfermagem, é exclusiva do enfermeiro^31^. Com a implementação do Processo de Enfermagem (PE) e de protocolos institucionais, é possível garantir uma assistência sistemática e segura^32^, alinhada à recomendação da ELSO de manter a proporção de 1:1 entre enfermeiro e paciente em ECMO^33^. É imperativo que os profissionais detenham conhecimentos, habilidades e atitudes fundamentais para o manejo desses pacientes^27^ ^,^ ^34^.

Por fim, é importante destacar que o hospital do estudo teve uma transição significativa nos registros. Até setembro de 2020, as anotações eram realizadas em prontuários físicos. Após esse período, foi implementado o PEP e, em 2023, o PE e a Sistematização da Assistência de Enfermagem (SAE), anteriormente baseada em “*Checklists*” físicos, tornou-se totalmente digital. Além disso, os demais membros da equipe multiprofissional de saúde também aderiram à mesma plataforma de registro, reduzindo o tempo destinado às anotações e permitindo mais tempo na assistência ao paciente.

A limitação deste estudo é que ele se baseia em registros que não contemplam desfechos a longo prazo. No entanto, durante a coleta das variáveis, os pesquisadores anotaram o número de telefone dos responsáveis pelo paciente, com o objetivo de, no futuro, avaliar por meio da telemedicina, as incapacidades e a qualidade de vida dos sobreviventes. Outra limitação foi a falta de informações completas em alguns prontuários especialmente nos registros físicos, como a modalidade e o tipo de ECMO utilizado. O fato de a pesquisa ser conduzida em apenas um centro limita generalizações com outros contextos. 

Além da pesquisa, os dados serão compartilhados com o hospital onde o estudo foi realizado, o que pode auxiliar a equipe multiprofissional na seleção tempestiva dos candidatos para o suporte extracorpóreo, potencializando as chances de sucesso da terapia-ponte e na otimização dos recursos do centro. O estudo evidencia que o uso da ECMO e as condições clínicas dos pacientes estão associados à ocorrência de eventos adversos, como complicações mecânicas e fisiológicas. Dada a escassez de estudos na faixa etária pediátrica submetida à ECMO, esta pesquisa traz contribuições significativas sobre o tema, representando um avanço do conhecimento científico para a área de saúde, na análise e divulgação de assuntos pouco explorados. 

Para a enfermagem, o conhecimento do perfil clínico-epidemiológico permite que enfermeiros(as) compreendam as particularidades desse público, adaptem a assistência com base na clínica do paciente e na modalidade da ECMO ofertada, fortalecendo a prática baseada em evidência e uma assistência segura e eficaz. 

## Conclusão

As complicações mecânicas na ECMO apresentaram associação significativa com o aumento do risco de mortalidade entre os pacientes analisados. O perfil clínico-epidemiológico evidencia a severidade do quadro clínico, marcada especialmente pela presença de instabilidade hemodinâmica e pela necessidade de intervenção cirúrgica. Além disso, a utilização prévia de drogas vasoativas e antibióticos pré-canulação reflete o agravamento cardíaco e infeccioso, contribuindo para a elevada mortalidade observada nessa população.

Este estudo apresenta contribuições para preencher lacunas na prática clínica em pacientes pediátricos submetidos a ECMO, servindo de base para a implementação de programas de educação permanente e para o desenvolvimento de linhas de cuidado específicas para esse público. Ressalta-se a importância de novas pesquisas, tanto clínicas quanto epidemiológicas, para uma caracterização mais abrangente dos pacientes pediátricos candidatos à ECMO, além do monitoramento a longo prazo dos sobreviventes.

## Declaração de Disponibilidade de Dados:

Todos os dados gerados ou analisados durante este estudo estão incluídos neste artigo publicado

# Translation: Perfil clínico-epidemiológico de pacientes pediátricos en oxigenación por membrana extracorpórea: experiencia de un centro^3^

**DOI:** 10.1590/1518-8345.7999.4779

**Authors:** Karleandro Pereira do Nascimento, Maria Veraci Oliveira Queiroz, Silvania Braga Ribeiro, Viviane Martins da Silva, Marcos Venícios de Oliveira Lopes, Thais Moreira de Sena

**Affiliations:** 1 Universidade Estadual do Ceará, Centro de Ciências da Saúde, Fortaleza, CE, Brazil.; 2 Scholarship holder at the Fundação Cearense de Apoio ao Desenvolvimento Científico e Tecnológico (FUNCAP), Brazil.; 3 Universidade Federal do Ceará, Faculdade de Farmácia, Odontologia e Enfermagem, Fortaleza, CE, Brazil.

**Keywords:** Enfermería, Pediatría, Registros Médicos, Enfermería de Práctica Avanzada, Cuidados Críticos, Oxigenación por Membrana Extracorpórea.

## Abstract

**Objetivo:**

analizar el perfil clínico-epidemiológico de pacientes pediátricos en soporte de oxigenación por membrana extracorpórea en un centro de referencia.

**Método:**

estudio transversal y retrospectivo, basado en el análisis de 108 historias clínicas, tanto electrónicas como físicas, de pacientes pediátricos de un hospital público. Se recolectaron datos sociodemográficos y clínicos mediante un instrumento desarrollado para esta investigación. Para el análisis, se utilizaron frecuencias absolutas y porcentajes, medidas de tendencia central y dispersión, prueba de normalidad de Shapiro-Wilk, prueba chi-cuadrado de Pearson, modelo de regresión logística con entrada Stepwise, análisis de regresión lineal múltiple y prueba de Durbin-Watson.

**Resultados:**

de los documentos analizados, 55,5% correspondían a pacientes de sexo femenino. En cuanto a los desenlaces clínicos, 76,1% aguardaban cirugía antes de la canulación, 69,3% utilizaron fármacos vasoactivos y 57,4% evolucionaron a fallecimiento. La oxigenación por membrana extracorpórea venoarterial de tipo central fue la modalidad más utilizada. La principal indicación para el soporte fue el síndrome de bajo gasto cardíaco. Los pacientes que desarrollaron complicaciones mecánicas en la oxigenación por membrana extracorpórea presentaron un riesgo estadísticamente mayor de fallecimiento (*odds ratio* de 2,8).

**Conclusión:**

las complicaciones mecánicas se asociaron con un mayor riesgo de mortalidad. El perfil clínico-epidemiológico evidencia la gravedad clínica, caracterizada por inestabilidad hemodinámica e indicación quirúrgica.

## Destacados

**(1)** La ECMO VA central fue la modalidad más utilizada en los pacientes pediátricos. **(2)** El síndrome de bajo gasto cardíaco fue la principal indicación para ECMO. **(3)** Las complicaciones mecánicas aumentaron en 2,8 veces el riesgo de fallecimiento. **(4)** Predominio de lactantes de sexo femenino con cardiopatías congénitas. **(5)** Mortalidad de 57,4% entre pacientes pediátricos sometidos a ECMO.

## Introducción

La oxigenación por membrana extracorpórea (ECMO) funciona como un soporte cardiopulmonar para cuadros de insuficiencia cardíaca (choque cardiogénico) y/o respiratoria (hipoxemia o hipercapnia) aguda y refractaria a los tratamientos médicos, pero con potencial de recuperación. Aunque se la mencione como una terapia, la ECMO debe entenderse como un soporte temporal, utilizado como puente para recuperación, toma de decisiones o para trasplante^1^ ^-^ ^2^. Su utilización en Brasil es relativamente reciente, habiendo sido reconocida mediante el Parecer n.º 42/2017 del *Conselho Federal de Medicina* como un procedimiento no experimental^3^. El país cuenta con 21 centros acreditados en la *Extracorporeal Life Support Organization* (ELSO), una entidad internacional que establece directrices sobre esta tecnología^4^.

La ECMO representa uno de los grandes avances de la medicina intensiva moderna para pacientes en estado crítico. La condición clínica del individuo y el órgano afectado determinan el tipo de soporte ofrecido. La modalidad venoarterial (VA) ofrece soporte cardíaco con función pulmonar preservada o no, teniendo como indicación más frecuente el fallo transitorio poscardiotomía o el choque cardiogénico. La modalidad venovenosa (VV) se utiliza para soporte respiratorio en pacientes con función cardíaca preservada, como en las neumonías^5^ ^-^ ^6^. La ECMO también abarca otras modalidades como la E-CPR (resucitación cardiopulmonar extracorpórea)^7^ y configuraciones combinadas como la VVV (veno-veno-venosa), VVA (veno-veno-arterial) y VAV (veno-arterial-venosa) que atienden situaciones más complejas^5^.

El primer éxito registrado del uso de un dispositivo de circulación extracorpórea ocurrió en una cirugía cardíaca en 1954. En 1972 se registró el primer uso de la ECMO como soporte terapéutico en el síndrome de dificultad respiratoria aguda (SDRA). El primer caso neonatal fue descrito en 1975 en una paciente con síndrome de aspiración meconial^8^. En el contexto pediátrico, las indicaciones más comunes consisten en la inestabilidad hemodinámica preoperatoria, el síndrome de bajo gasto cardíaco posoperatorio, la cardiopatía congénita crítica y la incapacidad para desvincularse del *bypass* cardiopulmonar. Considerando aisladamente la población pediátrica con cardiopatía congénita, el soporte extracorpóreo se indica comúnmente para la estabilización clínica pre o poscirugía y en el escenario de E-CPR^9^.

El tiempo durante el cual el paciente permanece en ECMO es variable, pudiendo extenderse por días o semanas. La interrupción de la terapia se relaciona con la mejoría de la condición clínica que justificó la necesidad del soporte, la ausencia de perspectiva de recuperación o la irreversibilidad del cuadro^5^. La ECMO en pacientes pediátricos presenta particularidades en el manejo respecto de los adultos, como la necesidad de ajustes específicos en los parámetros hemodinámicos y el mayor riesgo de complicaciones trombóticas y hemorrágicas^2^, aspectos que motivaron la realización de este estudio.

El análisis del perfil clínico-epidemiológico de pacientes pediátricos en ECMO amplía la comprensión del equipo multiprofesional sobre las complicaciones durante el soporte, el pronóstico y los desenlaces de la terapia-puente^2^ ^,^ ^9^. Los registros en las historias clínicas documentan los principales desafíos asociados al uso de la ECMO, los diagnósticos más prevalentes que justificaron su indicación, las conductas terapéuticas adoptadas, las respuestas al tratamiento y los desenlaces clínicos. Estos datos reflejan la evaluación continua del paciente, desde la canulación hasta el alta médica. Comprender estas relaciones contribuirá a la planificación de intervenciones que minimicen fallas en la asistencia y mejoren el manejo del soporte extracorpóreo.

Ante lo expuesto, se formuló la siguiente pregunta orientadora para este estudio: ¿Cuál es el perfil clínico-epidemiológico de pacientes pediátricos sometidos a ECMO en un centro de referencia? Para responderla, se estableció como objetivo analizar el perfil clínico-epidemiológico de pacientes pediátricos en soporte de oxigenación por membrana extracorpórea en un centro de referencia.

## Método

### Tipo de estudio

Estudio transversal, retrospectivo, unicéntrico de base cuantitativa^10^ realizado con historias clínicas electrónicas y físicas de pacientes pediátricos.

### Lugar y período de recolección

La recolección de datos fue realizada por dos investigadores en un hospital de referencia en cardioneumología de alta complejidad ubicado en Fortaleza, CE, Brasil, entre mayo y agosto de 2024. Esta institución atiende pacientes desde la franja etaria pediátrica hasta la edad adulta, abarcando las regiones Norte y Nordeste del país e integrando uno de los mayores complejos de salud totalmente conveniados por el Sistema Único de Salud (SUS). El hospital cuenta con ocho unidades de cuidados intensivos (UCI), siendo dos destinadas a la atención de niños con cardiopatías congénitas. Entre ellas, una UCI es exclusiva para el posoperatorio de cirugía cardíaca y para pacientes en ECMO, disponiendo de ocho camas. El estudio fue conducido en esa unidad.

### Población, criterios de selección y muestra

La población estuvo compuesta por todos los pacientes pediátricos sometidos a ECMO entre julio de 2012, año y mes de inicio del programa, y abril de 2024, totalizando 12 años de experiencia en asistencia extracorpórea en ese centro. Se analizaron los registros de todos los pacientes canulados en ese período, admitidos en la UCI posoperatoria de cirugía cardíaca pediátrica del hospital. La investigación utilizó una muestra no probabilística por conveniencia, resultando en un total de 108 individuos pediátricos; no hubo canulación neonatal en el período analizado. Como la muestra incluyó todas las historias clínicas disponibles, ningún documento fue excluido, no habiendo, por tanto, criterio de exclusión.

### Recolección de datos y variables del estudio

Las historias clínicas físicas fueron localizadas en el archivo de la Secretaría de Salud del Estado de Ceará y transportadas al centro de recolección para la extracción de la información. Las historias clínicas electrónicas fueron accedidas directamente en las computadoras del servicio de origen, garantizando la obtención de los datos de manera sistemática y confidencial. Las historias clínicas físicas fueron incluidas, dado que al inicio de la implementación de la ECMO en el hospital del estudio no había registros electrónicos en salud. La lectura de las historias clínicas se realizó en una sala del Departamento de Registro y Archivos Médicos del hospital del estudio.

Las variables evaluadas fueron: sexo, edad, peso, diagnóstico primario, indicación para el soporte, uso de fármacos vasoactivos antes de la ECMO, número de días de ventilación mecánica invasiva antes de la instalación del soporte, ocurrencia de paro cardiorrespiratorio (PCR) previo, poscardiotomía como indicación, lugar físico de la canulación, modalidad de la ECMO, duración de la asistencia extracorpórea, uso de antibióticos (cuando aplicable), complicaciones mecánicas y fisiológicas y desenlace del paciente.

Se entiende por complicaciones mecánicas: falla de la membrana oxigenadora; falla de la bomba; rotura de los circuitos; problemas con las cánulas (mal posicionamiento u obstrucción por coágulos); y embolia gaseosa. Las complicaciones fisiológicas incluyen: hemorragia; lesión renal aguda y/o necesidad de terapia de sustitución renal; lesión del sistema nervioso central; desequilibrios hidroelectrolítico y metabólico; complicaciones cardíacas, incluyendo el uso de agentes inotrópicos/vasopresores; y otras complicaciones, incluyendo infecciones, neumotórax, hemólisis y lesión por presión^11^.

### Tratamiento y análisis de los datos

La información recolectada por los investigadores fue organizada en una hoja de cálculo de *Excel* almacenada en una unidad de almacenamiento. Los datos fueron estructurados con base en un instrumento de recolección desarrollado específicamente para esta investigación. La lectura de las historias clínicas siguió las categorías de análisis establecidas en el instrumento, respetando el código de ética de las profesiones involucradas y los modos de registro adoptados por los profesionales.

Los datos fueron analizados por medio de frecuencias absolutas y porcentajes para variables cualitativas, mientras que para variables cuantitativas se presentaron las medidas de tendencia central media y mediana, y de dispersión, desviación estándar e intervalo intercuartílico. Además, se aplicó la prueba de Shapiro-Wilk para verificación de adherencia a la distribución normal. La prueba de chi-cuadrado de Pearson fue aplicada para verificar la asociación entre las variables clínicas y el fallecimiento.

Para identificar las características que influenciaban el tipo de ECMO, se aplicó un modelo de regresión logística con entrada Stepwise. Para la evaluación de los factores que influenciaban el tiempo de ECMO, se utilizó el análisis de regresión lineal múltiple con verificación de ajuste por el coeficiente de determinación (R^2^). La autocorrelación de los residuos fue analizada mediante la prueba de Durbin-Watson.

### Aspectos éticos

Todos los requisitos éticos y legales para investigación con seres humanos fueron atendidos, con aprobación del Comité de Ética en Investigación de la institución proponente, conforme al parecer número 6.050.790 y CAAE número 69346823.4.0000.5039. Se obtuvo el término de anuencia del hospital donde el estudio fue conducido y la autorización de los gestores de la UCI posoperatoria cardíaca pediátrica. La información recolectada fue tratada con confidencialidad, asegurando el anonimato de los pacientes y la confidencialidad del lugar de la investigación, en conformidad con las normas reguladoras^12^.

## Resultados

A lo largo de un período de doce años (2012-2024), 108 pacientes pediátricos fueron sometidos al soporte de ECMO. La muestra abarcó la totalidad de los documentos disponibles. Sin embargo, se identificó ausencia de datos en algunas variables por falta de registros, sobre todo en las historias clínicas físicas, lo que explica la diferencia en los valores totales presentados en las Tablas 2 y 3.

De los documentos analizados, la mayoría correspondía a pacientes de sexo femenino (60; 55,5%). Los resultados demuestran una edad mediana de nueve meses de los individuos, con una mediana de peso de siete kilogramos. De las 108 historias clínicas, 52 provenían del archivo físico, procedentes del acervo y custodia de la Secretaría de Salud del Estado de Ceará, mientras que 56 pertenecían a la historia clínica electrónica del paciente (PEP), programa iniciado en el hospital en el año 2020, conforme la Tabla 1.

**Tabla 1:** Caracterización del perfil sociodemográfico de los pacientes pediátricos sometidos a ECMO* (n = 108). Fortaleza, CE, Brasil, 2025

Variables	n^†^		%^‡^		
Sexo					
Femenino	60		55,56		
Masculino	48		44,44		
Variables	Media	Desviación estándar	Mediana	IIQ^§^	Valor p^||^
Edad (meses)	38,0	63,5	9,0	35,0	<0,001
Peso (kilo)	15,1	20,1	7,0	9,0	<0,001

*ECMO = Oxigenación por membrana extracorpórea; ^†^n = Participantes; ^‡^% = Porcentaje; ^§^IIQ = Intervalo intercuartílico; ^||^Valor p = Hipótesis nula

La Tabla 2 presenta el desenlace clínico de los pacientes pediátricos, evidenciando que más de la mitad aguardaba cirugía debido a la gravedad de la cardiopatía congénita (64; 76,1%) antes de la canulación o utilizaba fármacos inotrópicos o vasopresores (52; 69,3%). La modalidad más utilizada fue la ECMO VA con predominio del tipo central. El soporte se instaló mayoritariamente en el centro quirúrgico del hospital de origen y la principal indicación para el uso del soporte fue el síndrome de bajo gasto cardíaco.

**Tabla 2:** Desenlace clínico de los pacientes pediátricos, modalidad y tipo de ECMO*, lugar físico de la instalación e indicación del soporte (n = 108). Fortaleza, CE, Brasil, 2025

Variables				n^†^	%^‡^
Clínica					
Corrección quirúrgica pre-ECMO*				64	76,19
Complicaciones fisiológicas				64	73,56
uso de antibióticos en el día de la instalación				64	73,56
Fármaco vasoactivo pre-ECMO*				52	69,33
Fallecimiento				54	57,45
Complicaciones mecánicas				41	47,13
PCR^§^ pre-ECMO*				28	36,84
Modalidad de la ECMO*					
Venoarterial				74	92,50
Venovenosa				6	7,50
Tipo de ECMO*			
Central				52	65,00
Periférica				28	35,00
Lugar de instalación					
Centro quirúrgico del hospital de origen				45	56,25
Unidad de cuidados intensivos del hospital de origen				33	41,25
Otro hospital				2	2,50
Indicación de la ECMO*					
Síndrome de bajo gasto cardíaco				22	26,83
Choque cardiogénico				13	15,85
Crisis de hipertensión pulmonar/hipoxemia				13	15,85
Síndrome de dificultad respiratoria aguda/coronavirus				9	10,98
Otras causas				8	9,76
Pos-circulación extracorpórea				5	6,10
Disfunción de ventrículo derecho				5	6,10
Neumonía/Choque séptico				4	4,88
Variables	Media	Desviación estándar	Mediana	IIQ^||^	Valor p^¶^
Ventilación Mecánica (días)	3,5	4,3	1,0	3,2	<0,001
Tiempo de oxigenación por membrana extracorpórea	9,5	9,8	6,0	6,0	<0,001

*ECMO = Oxigenación por membrana extracorpórea; ^†^n = Participantes; ^‡^% = Porcentaje; ^§^PCR = Paro cardiorrespiratorio; ^||^IIQ = Intervalo intercuartílico; ^¶^Valor p = Hipótesis nula

Los niños que presentaron complicaciones mecánicas durante la ECMO tuvieron una probabilidad casi tres veces mayor de evolucionar a fallecimiento, en comparación con aquellos que no desarrollaron tales complicaciones (*odds ratio* de 2,8). Es importante resaltar que la muestra comprendió todos los documentos disponibles; no obstante, algunas variables presentaron datos incompletos, principalmente en las historias clínicas físicas, lo que justifica la variación total en la Tabla 3.

**Tabla 3:** Complicación mecánica y fallecimiento de los pacientes pediátricos en ECMO* (n = 87). Fortaleza, CE, Brasil, 2025

Complicación mecánica	Fallecimiento		Total	χ^2†^	Valor p^‡^	***Odds Ratio* (IC - 95%** ^§^)
	Sí	No				
Sí	28	13	41	5,40	0,010	2,80 (1,16 - 6,74)
No	20	26	46			
Total	48	39	87			

*ECMO = Oxigenación por membrana extracorpórea; ^†^χ2 = Chi-cuadrado; ^‡^Valor p = Hipótesis nula; ^§^IC-95% = Intervalo de confianza

En la Tabla 4, el modelo de regresión logística mostró una reducción en la probabilidad de uso de ECMO periférica entre los niños que no presentaron PCR pre-ECMO y fueron sometidos al procedimiento en el centro quirúrgico en comparación con aquellos atendidos en la UCI. Por otro lado, la probabilidad de ECMO central fue mayor entre los niños de la UCI y con PCR pre-ECMO.

En la Tabla 5, el modelo de regresión lineal para la duración de la ECMO demostró que el tiempo de soporte era mayor entre pacientes del sexo femenino y entre quienes no habían sido sometidos a corrección quirúrgica.

**Tabla 4:** Modelo de regresión logística multivariada para tipo de ECMO* (periférica) (n = 108). Fortaleza, CE, Brasil, 2025

Variables	Coef^†^	EP^‡^	z^§^	Wald^||^	gl^¶^	Valor p**	*Odds Ratio* ^*††*^	IC-95%^‡‡^
Intercepto	1,05	0,73	1,43	2,04	1	0,153		
Edad (meses)	0,03	0,01	3,10	9,62	1	0,002	1,03	1,01 - 1,04
PCR^§§^ pre ECMO* (No)	-1,89	0,78	-2,43	5,92	1	0,015	0,15	0,03 - 0,69
Instalación: hospital de origen	-2,76	0,81	-3,40	11,53	1	<0,001	0,06	0,01 - 0,31
Instalación: otro centro	-0,43	1,65	-0,26	0,07	1	0,796	0,65	0,03 - 16,50

*ECMO = Oxigenación por membrana extracorpórea; ^†^Coef = Coeficiente; ^‡^EP = Error estándar; ^§^z = Estadístico; ^||^Wald = Prueba de Wald; ^¶^gl = Grados de libertad; **Valor p = Hipótesis nula; ^††^ *Odds ratio* = Razón de probabilidades; ^‡‡^IC-95% = Intervalo de confianza; ^§§^PCR = Paro cardiorrespiratorio

**Tabla 5:** Modelo de regresión lineal múltiple para tiempo de ECMO*. Fortaleza, CE, Brasil, 2025

Variables				Coef^†^	EP^‡^	t^§^	Valor p^||^
Intercepto				5,34	1,78	3,00	0,004
Sexo (Femenino)				4,45	2,17	2,05	0,044
Corrección quirúrgica pre ECMO* (No)				6,39	2,44	2,62	0,011
Anova			SQ^¶^	gl**	QM^††^	F^‡‡^	Valor p^||^
Regresión			896,2	2	448,1	5,18	0,008
Residuo			6232,4	72	86,6		
Total			7128,7	74			
Ajuste			R²^§§^	R²aj^||||^	Autocorr^¶¶^	DW***	Valor p^||^
			0,126	0,101	-0,041	2,08	0,755

*ECMO = Oxigenación por membrana extracorpórea; ^†^Coef = Coeficiente; ^‡^EP = Error estándar; ^§^t = Estadístico t; ^||^Valor p = Hipótesis nula; ^¶^SQ = Suma de cuadrados; **gl = Grados de libertad; ^††^QM = Cuadrado medio; ^‡‡^F = Estadístico F; ^§§^R² = Coeficiente de determinación; ^||||^R²aj = Coeficiente de determinación ajustado; ^¶¶^Autocorr. = Autocorrelación; ***DW = Prueba de Durbin-Watson

## Discusión

Por medio de este estudio fue posible analizar el perfil clínico-epidemiológico de pacientes pediátricos con ECMO en un hospital de referencia en cardioneumología en el Estado de Ceará. Se considera esta terapia-puente cuando la insuficiencia de la función pulmonar y/o cardíaca aguda representa un riesgo de mortalidad superior a 50%, siendo altamente indicada cuando la probabilidad de muerte alcanza cerca de 80%, a pesar del tratamiento convencional^13^ ^-^ ^14^. En consonancia con lo descrito en la literatura^15^ ^-^ ^16^, los datos de esta investigación señalan un predominio en el uso de la ECMO en pacientes lactantes de sexo femenino, siendo más frecuentemente indicada para los casos de cardiopatías congénitas. Los hallazgos están en consonancia con un estudio previo que indica una alta prevalencia de la ECMO en ese grupo etario^16^.

La ELSO recomienda criterios de selección en casos de falla respiratoria y de falla cardíaca con o sin disfunción pulmonar asociada, para los pacientes pediátricos que serán sometidos a la terapia^14^ ^,^ ^17^. Las decisiones deben tomarse considerando la individualidad de cada paciente, con base en el conocimiento de la condición clínica, la experiencia del centro, el consenso del equipo multiprofesional y la consulta a pie de cama^14^. En este estudio, la indicación ocurrió dentro de los criterios establecidos internacionalmente, con destaque para la duración de la ventilación mecánica, que presentó una media de 3,5 días hasta el momento de la canulación. Se observa que las tasas de supervivencia son mayores cuando el período de ventilación mecánica es inferior a 14 días pre-ECMO^18^ ^-^ ^19^.

El análisis de los datos revela que los pacientes ya utilizaban fármacos vasoactivos antes del inicio de la ECMO, evidenciando un cuadro de inestabilidad hemodinámica previo a la canulación. Además, una proporción significativa de los individuos presentó PCR antes del inicio de la terapia. Aunque los fármacos vasoactivos pueden actuar como rescate hemodinámico del paciente crítico, su uso está asociado a peores desenlaces, como nefrotoxicidad renal e hipoperfusión tisular, mayor tiempo de permanencia en la UCI y aumento de la mortalidad^20^. Durante la ECMO, estos medicamentos generalmente tienen sus dosis reducidas de forma gradual hasta la suspensión, con la necesidad de mantenimiento o retirada evaluada caso a caso^16^.

El uso de fármacos vasoactivos refleja el agravamiento clínico de los pacientes con cardiopatías congénitas que aguardaban cirugías paliativas, correctivas o procedimientos hemodinámicos. En cerca de un tercio de los casos, la PCR fue la principal indicación para el uso de la ECMO. En esa situación, la instalación de ECMO VA puede recomendarse cuando el paciente no responde a las maniobras convencionales de resucitación cardiopulmonar (RCP), procedimiento conocido como E-CPR^21^ ^-^ ^22^. Restringida a la PCR intrahospitalaria, la E-CPR debe instituirse durante las compresiones cardíacas o dentro de 20 minutos tras el retorno de la circulación espontánea sin compresiones continuas. Su utilización está asociada a desenlaces neurológicos menos favorables en comparación con la ECMO empleada para otras indicaciones^22^.

En el presente estudio, los pacientes sometidos a canulación, sin la PCR como criterio, presentaron complicaciones fisiológicas, como sangrado, lesión por presión, infección y desequilibrio hidroelectrolítico y metabólico, tendencia igualmente reportada en un estudio previo^11^. También se observaron en esta investigación complicaciones mecánicas como formación de coágulos en las cánulas, decanulación accidental y falla del oxigenador, en consonancia con reportes de un estudio anterior^18^. Se observó que la enfermedad de base incrementó el riesgo de sangrado y de tromboembolismo, asociado al uso del anticoagulante continuo y a la interfaz no biológica del circuito, dato semejante al encontrado en otra investigación^16^. Cabe añadir que el soporte VA de predominio central, descrito en la mayoría de los casos, acarrea perjuicios en el desempeño cardíaco, reducción del flujo coronario, menor transporte de oxígeno al cerebro y a los órganos vitales y aumento de la resistencia vascular pulmonar^23^.

El soporte VV está asociado a menor riesgo de lesión neurológica y mortalidad, siendo recomendado como modalidad inicial para los casos de insuficiencia respiratoria en pediatría^14^. En esa condición, la neumonía bacteriana figura como una causa frecuente de ECMO en ese grupo etario^24^.

En este estudio, las principales indicaciones para el soporte extracorpóreo fueron el síndrome de bajo gasto cardíaco, el choque cardiogénico y la crisis de hipertensión pulmonar/hipoxemia, con una tasa de mortalidad superior a la mitad de la población analizada. Dado el predominio de causas cardíacas, se especula que factores como la utilización de ECMO en la modalidad VA, el uso previo de inotrópicos y vasopresores, la indicación del soporte en el momento oportuno, la enfermedad subyacente e incluso aspectos demográficos, como la franja etaria, pueden haber influenciado la mortalidad observada.

Los estudios señalan que la indicación basada en criterios bien definidos contribuye al éxito de la terapia^13^ ^,^ ^15^ ^,^ ^25^. El objetivo de la ECMO no es curativo, sino ofrecer soporte al paciente crítico cuando las opciones convencionales se muestran ineficaces, aumentando sus posibilidades de supervivencia^17^. A diferencia de otros métodos, como el dispositivo de asistencia ventricular (VAD), que requiere instalación en un ambiente estéril, la ECMO puede implantarse a pie de cama^26^, como ocurrió en este estudio.

Aunque el soporte extracorpóreo sea una estrategia vital para los pacientes en estado crítico, la presencia de múltiples conectores y puntos de acceso aumenta el riesgo de complicaciones y desafíos operativos, como sangrado y formación de trombos en el circuito, ambos asociados a una alta tasa de mortalidad^23^ ^,^ ^25^. En este estudio, las principales causas de fallecimiento fueron fallo multiorgánico, PCR y choque séptico.

La conexión prolongada del organismo a la superficie de polivinilo de las cánulas, el uso de múltiples dispositivos médicos y la condición clínica del individuo representan factores de riesgo para infecciones. El manejo del paciente por el equipo multiprofesional, utilizando técnicas asépticas, es esencial para minimizar este riesgo^26^ ^-^ ^27^. Aunque la literatura no presenta datos concluyentes que asocien el lugar de instalación de la ECMO con el aumento del riesgo de infección, es mandataria la adopción de la asepsia, sobre todo cuando la canulación ocurre en la UCI^28^. Los datos muestran que los pacientes ya estaban en uso de antibióticos antes de la canulación, lo que refleja la complejidad del cuadro clínico-infeccioso. El tiempo medio de utilización de la ECMO fue de 9,5 días, resultados semejantes a los de la literatura^15^ ^-^ ^16^.

A pesar de los avances en el manejo del dispositivo, los riesgos asociados al uso prolongado del soporte permanecen inevitables^29^. Se observó que los niños que desarrollaron complicaciones mecánicas presentaron un riesgo de fallecimiento significativamente mayor, siendo casi tres veces superior al de quienes no tuvieron esas complicaciones. Las más comunes incluyen mala posición de las cánulas, decanulación accidental, formación de coágulos en el oxigenador y la retirada inadvertida de las cánulas por pacientes despiertos^11^ ^,^ ^29^ ^-^ ^30^. La vigilancia continua y la respuesta rápida ante eventos adversos con ECMO son indispensables. Aunque raras, esas complicaciones pueden causar daños irreversibles al paciente o incluso el fallecimiento^30^.

En este estudio, la decanulación accidental ocurrió en un paciente durante el cambio de decúbito, realizado por dos profesionales para prevenir la lesión por presión. Sin embargo, la imagen radiológica ya indicaba un alto riesgo para ese evento adverso, que no fue identificado previamente por el equipo. Considerada catastrófica, la decanulación accidental puede provocar sangrado masivo y entrada de aire en el circuito^15^ ^,^ ^30^. Este fue el evento adverso más grave de la muestra, lo que motivó la implementación de capacitaciones y estrategias preventivas. El paciente evolucionó a fallecimiento debido a la enfermedad subyacente, y no por la decanulación, en otra unidad hospitalaria.

Este estudio revela una menor probabilidad de utilización de ECMO periférica en pacientes sin PCR antes de la canulación y que habían pasado por cirugía cardíaca. El soporte fue más frecuentemente necesario en pacientes internados en la UCI con PCR previa y que no habían realizado cirugía. Esos datos destacan la importancia de intervenciones oportunas, ya que los niños con cardiopatías congénitas, que compusieron casi toda la muestra, necesitan procedimientos quirúrgicos en edades específicas, conforme a la fisiología de la enfermedad de base^15^ ^,^ ^20^. Debido a la gravedad de esas condiciones, es común el uso de la ECMO en pacientes con cardiopatías congénitas críticas^16^.

Aunque el manejo del soporte extracorpóreo involucra a un equipo multiprofesional, la ELSO destaca el papel de la enfermería en ese proceso. Siendo una terapia de alta complejidad y elevado costo, la responsabilidad por la asistencia directa a los pacientes en ECMO, dentro del equipo de enfermería, es exclusiva del enfermero^31^. Con la implementación del Proceso de Enfermería (PE) y de protocolos institucionales, es posible garantizar una asistencia sistemática y segura^32^, alineada con la recomendación de la ELSO de mantener la proporción de 1:1 entre enfermero y paciente en ECMO^33^. Es imperativo que los profesionales detenten conocimientos, habilidades y actitudes fundamentales para el manejo de esos pacientes^27^ ^,^ ^34^.

Por último, es importante destacar que el hospital del estudio tuvo una transición significativa en los registros. Hasta septiembre de 2020, las anotaciones eran realizadas en historias clínicas físicas. Tras ese período, se implementó el PEP y, en 2023, el Proceso de Enfermería (PE) y la Sistematización de la Atención de Enfermería (SAE), anteriormente basada en “*Checklists*” físicos, se volvió totalmente digital. Además, los demás miembros del equipo multiprofesional de salud también adhirieron a la misma plataforma de registro, reduciendo el tiempo destinado a las anotaciones y permitiendo más tiempo en la atención al paciente.

La limitación de este estudio es que se basa en registros que no contemplan desenlaces a largo plazo. No obstante, durante la recolección de las variables, los investigadores anotaron el número de teléfono de los responsables del paciente, con el objetivo de, en el futuro, evaluar, por medio de la telemedicina, las discapacidades y la calidad de vida de los sobrevivientes. Otra limitación fue la falta de información completa en algunas historias clínicas, especialmente en los registros físicos, como la modalidad y el tipo de ECMO utilizado. El hecho de que la investigación sea conducida en solo un centro limita generalizaciones con otros contextos.

Además de la investigación, los datos serán compartidos con el hospital donde se realizó el estudio, lo que puede auxiliar al equipo multiprofesional en la selección oportuna de los candidatos para el soporte extracorpóreo, potenciando las probabilidades de éxito de la terapia-puente y la optimización de los recursos del centro. El estudio evidencia que el uso de la ECMO y las condiciones clínicas de los pacientes están asociados a la ocurrencia de eventos adversos, como complicaciones mecánicas y fisiológicas. Dada la escasez de estudios en la franja etaria pediátrica sometida a ECMO, esta investigación aporta contribuciones significativas sobre el tema, representando un avance del conocimiento científico para el área de la salud, en el análisis y divulgación de asuntos poco explorados.

Para la enfermería, el conocimiento del perfil clínico-epidemiológico permite que enfermeros(as) comprendan las particularidades de ese público, adapten la asistencia con base en la clínica del paciente y en la modalidad de ECMO ofrecida, fortaleciendo la práctica basada en evidencia y una asistencia segura y eficaz.

## Conclusión

Las complicaciones mecánicas en la ECMO presentaron una asociación significativa con el incremento del riesgo de mortalidad entre los pacientes analizados. El perfil clínico-epidemiológico evidencia la gravedad del cuadro clínico, marcada especialmente por la presencia de inestabilidad hemodinámica y por la necesidad de intervención quirúrgica. Además, el uso previo de fármacos vasoactivos y de antibióticos antes de la canulación refleja el agravamiento cardíaco e infeccioso, lo que contribuye a la elevada mortalidad observada en esta población.

Este estudio aporta contribuciones para subsanar brechas en la práctica clínica en pacientes pediátricos sometidos a ECMO, sirviendo de base para la implementación de programas de educación permanente y para el desarrollo de líneas de cuidado específicas para este público. Se resalta la importancia de nuevas investigaciones, tanto clínicas como epidemiológicas, para una caracterización más amplia de los pacientes pediátricos candidatos a ECMO, además del seguimiento a largo plazo de los sobrevivientes.

## Declaración de Disponibilidad de Datos:

Todos los datos generados o analizados durante este estudio están incluidos en este artículo publicado
